# Supplementary material for: Permeabilization-free en bloc immunohistochemistry for correlative microscopy
Source: eLife. 2021 May 13;10:e63392. doi: 10.7554/eLife.63392 (PMC8118656; doi:10.7554/eLife.63392)
Supplement: Figure 1—source data 1. — Primary and secondary antibodies that successfully labeled protein targets throughout the depth of 300-μm-thick acute extracellular space-preserved brain slices. [file elife-63392-fig1-data1.pdf]

**Primary Antibody**	**Company and Cat #**	**Stock Concentration**	**Concentration**	**Target**
Anti-calbindin (CB)	Synaptic Systems 214 005	1 mg/mL	1:100	Calcium-binding protein
Anti-calretinin (CR)	Sigma C7479	0.8 mg/mL	1:50	Calcium-binding protein
Anti-tyrosine hydroxylase (TH)	EMD Millipore MAB318-AF488	0.5 mg/mL	1:100	Enzyme
Anti-choline acetyltransferase (ChAt)	EMD Millipore AB144P	0.5 mg/mL	1:100	Enzyme
Anti-synaptophysin (Syn) (Alexa Fluor-594)	Abcam ab206868	0.5 mg/mL	1:50	Presynaptic protein
Anti-synaptophysin (Syn) (Alexa Fluor-488)	Abcam ab196379	0.5 mg/mL	1:50	Presynaptic protein
Anti-Homer 1	Synaptic Systems 160 003	1 mg/mL	1:50	Postsynaptic protein
Anti-NeuN	Abcam ab190195	0.5 mg/mL	1:50	Neuronal marker
Anti-GFP	ThermoFisher G10362	0.2 mg/mL	1:50	Marker protein

**Secondary Antibody**	**Company and Cat #**	**Stock Concentration**	**Concentration**
Alexa Fluor 594 $F(ab')_2$ fragment donkey anti-rabbit	Jackson ImmunoResearch 711-586-152	1.5 mg/mL	1:100
DyLight 594 donkey anti-goat	Abcam ab96937	0.5 mg/mL	1:100
DyLight 405 goat anti-guinea pig $F(ab')_2$ fragment specific	Jackson ImmunoResearch 106-475-006	1.5 mg/mL	1:25
HRP Donkey anti-rabbit	Abcam ab205722	2 mg/mL	1:50
