# Supplementary material for: Permeabilization-free en bloc immunohistochemistry for correlative microscopy
Source: eLife. 2021 May 13;10:e63392. doi: 10.7554/eLife.63392 (PMC8118656; doi:10.7554/eLife.63392)
Supplement: Supplementary file 1. — Incubation solutions, durations, and temperatures are reported along with reference to relevant supplementary figures. The duration of antibody incubation was varied dependent on section thickness. Asterisk (*) indicates sodium phosphate buffer (PB) concentration should be varied to achieve the desired extracellular space (ECS) volume fraction in a tissue-dependent manner. ACSF: artificial cerebral spinal fluid; PFA: paraformaldehyde; GA: glutaraldehyde; Ab: antibody; CB: sodium cacodylate buffer; NIRB: near-infrared branding; RT: room temperature; o/n: overnight. [file elife-63392-supp1.pdf]

Supplementary File 1

**Solution**	**Duration**	**Temp.**		**Relevant Supplemental Figures**
ACSF	~10 min	4° C	Dissection, acute sectioning	
4% PFA + 0.005% GA in 0.175 M PB*, pH 7.4	4 - 16 hr	4° C	First fixation	Figure 1-figure supplement 2 - Optimization of fixation parameters<br>Figure 1-figure supplement 3 - Optimal duration of primary fixation
0.05 M glycine in 0.175 M PB, pH 7.4	o/n	RT	Glycine rinse	
0.175 M PB, pH 7.4	4 hr	RT	Rinse	
0.17 M NaCl + 0.01 M PB + 1° Ab	2 - 7 days	RT	Primary antibody	Figure 1-figure supplement 4 - Effect of serum blocking<br>Figure 1-figure supplement 7 - Antibodies tested
0.175 M PB, pH 7.4	o/n	RT	Rinse	
0.17 M NaCl + 0.01 M PB + 2° Ab	2 - 7 days	RT	Secondary antibody	Figure 1-figure supplement 5 - Effect of prolonged antibody incubation
0.175 M PB, pH 7.4	4 hr - o/n	RT	Rinse	
2% PFA in 0.175 M PB, pH 7.4	2 hr	RT	Second fixation	
0.05 M glycine in 0.175 M PB, pH 7.4	4 hr	RT	Glycine rinse	
0.175 M PB, pH 7.4	4 hr	RT	Rinse	
20 → 100% fructose	20 - 48 hr	RT	SeeDB refractive index matching	Figure 1-figure supplement 6- Effect of SeeDB clearing
100% wt/vol or 80.2% wt/wt fructose	< 1 day	RT	2P imaging & NIRB	
0.175 M PB, pH 7.4	12 hr	RT	Return tissue to PB	
0.15 M CB, pH 7.4	2 hr	4° C	Rinse	
2% GA in 0.15 M CB, pH 7.4	2 hr	4° C	Third fixation	
0.15 M CB, pH 7.4	o/n	4° C	Rinse	
	2 days		EM staining/embedding	
			EM imaging	
